# (*E*)-1-(2-Thien­yl)-3-(2,4,5-trimethoxy­phen­yl)prop-2-en-1-one

**DOI:** 10.1107/S1600536808021375

**Published:** 2008-07-16

**Authors:** Hoong-Kun Fun, Samuel Robinson Jebas, P. S. Patil, S. M. Dharmaprakash

**Affiliations:** a X-ray Crystallography Unit, School of Physics, Universiti Sains Malaysia, 11800 USM, Penang, Malaysia; b Department of Studies in Physics, Mangalore University, Mangalagangotri, Mangalore 574 199, India

## Abstract

In the title mol­ecule, C_16_H_16_O_4_S, the enone fragment, thio­phene ring and benzene ring are individually essentially planar. The thio­phene ring is disordered over two sites, corresponding to a rotation of approximately 180° about the single C—C bond to which it is attached. The approximate ratio of occupancies for the major and minor components is 0.872 (2):0.128 (2). The major component of the thio­phene ring and the benzene ring are twisted from each other by 13.92 (19)°. An intra­molecular C—H⋯O hydrogen bond generates an *S*(5)*S*(5) ring motif. The crystal structure is stabilized by inter­molecular C—H⋯O hydrogen-bonding inter­actions. In addition, a π–π stacking inter­action, with a centroid–centroid distance of 3.852 (2) Å, and short S⋯O [2.9378 (12) Å] and O⋯O [2.5811 (16) Å] contacts are observed.

## Related literature

For related literature, see: Chantrapromma *et al.* (2005 ▶, 2006 ▶); Fun *et al.* (2006 ▶); Patil, Fun *et al.* (2007 ▶); Patil, Dharmaprakash *et al.*(2007 ▶). For bond-length data, see: Allen *et al.* (1987 ▶); Patil *et al.* (2006 ▶). For graph-set analysis of hydrogen bonding, see: Bernstein *et al.* (1995 ▶).
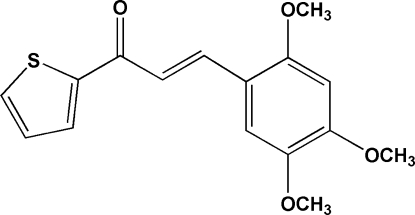


## Experimental

### 

#### Crystal data

C_16_H_16_O_4_S
*M* *_r_* = 304.35
Monoclinic, 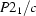

*a* = 7.5391 (1) Å
*b* = 7.9225 (1) Å
*c* = 24.3399 (3) Å
β = 97.021 (1)°
*V* = 1442.88 (3) Å^3^
*Z* = 4
Mo *K*α radiation
μ = 0.24 mm^−1^
*T* = 100.0 (1) K
0.25 × 0.22 × 0.14 mm

#### Data collection

Bruker SMART APEXII CCD area-detector diffractometer
Absorption correction: multi-scan (*SADABS*; Bruker, 2005 ▶) *T*_min_ = 0.942, *T*_max_ = 0.967
33027 measured reflections
4256 independent reflections
3174 reflections with *I* > 2σ(*I*)
*R*_int_ = 0.063

#### Refinement

*R*[*F* ^2^ > 2σ(*F* ^2^)] = 0.045
*wR*(*F* ^2^) = 0.120
*S* = 1.07
4256 reflections
211 parameters
120 restraints
H-atom parameters constrained
Δρ_max_ = 0.41 e Å^−3^
Δρ_min_ = −0.22 e Å^−3^

### 

Data collection: *APEX2* (Bruker, 2005 ▶); cell refinement: *APEX2*; data reduction: *SAINT* (Bruker, 2005 ▶); program(s) used to solve structure: *SHELXTL* (Sheldrick, 2008 ▶); program(s) used to refine structure: *SHELXTL*; molecular graphics: *SHELXTL*; software used to prepare material for publication: *SHELXTL* and *PLATON* (Spek, 2003 ▶).

## Supplementary Material

Crystal structure: contains datablocks global, I. DOI: 10.1107/S1600536808021375/lh2658sup1.cif

Structure factors: contains datablocks I. DOI: 10.1107/S1600536808021375/lh2658Isup2.hkl

Additional supplementary materials:  crystallographic information; 3D view; checkCIF report

## Figures and Tables

**Table 1:** Hydrogen-bond geometry (Å, °)

*D*—H⋯*A*	*D*—H	H⋯*A*	*D*⋯*A*	*D*—H⋯*A*
C2—H2*A*⋯O1^i^	0.93	2.55	3.363 (4)	146
C7—H7*A*⋯O1	0.93	2.52	2.8416 (18)	101
C7—H7*A*⋯O4	0.93	2.39	2.7477 (19)	103
C16—H16*B*⋯O4^ii^	0.96	2.56	3.2952 (19)	133

Symmetry codes: (i) 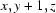; (ii) 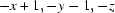.

**Fig. 1.**
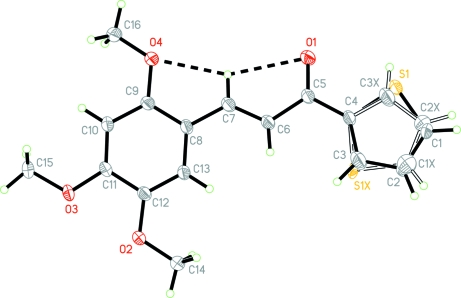
The molecular structure of the title compound, showing 50% probability displacement ellipsoids and the atom numbering scheme. Open bonds indicate the minor disorder component. Dashed lines indicate hydrogen bonds.

**Fig. 2.**
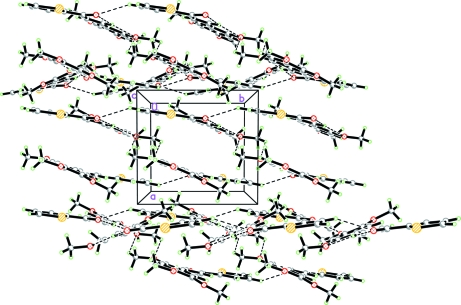
The crystal packing of the major component of the title compound,viewed along the c axis. Hydrogen bonds are shown as dashed lines.

**Fig. 3.**
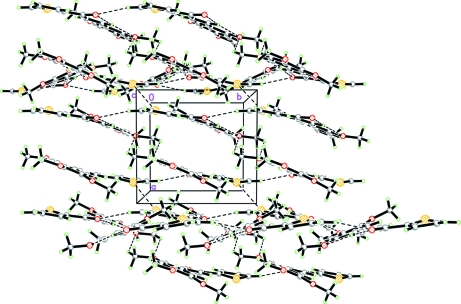
The crystal packing of the minor component of the title compound, viewed along the c axis. Hydrogen bonds are shown as dashed lines.

## supplementary crystallographic information

### Comment

The title compound (I) has been synthesized as part of our crystallographic studies on nonlinear optical materials (Chantrapromma *et al.*, 2005, 2006; Fun *et al.*, 2006; Patil, Fun *et al.*, 2007; Patil, Dharmaprakash *et al.*, 2007). We report herein the crystal structure of the title compound, (I).

In the title molecular structure (I), the thiophene ring is disordered over two sites (atoms of the minor occupancy component are labelled with the suffix *X*), corresponding to a rotation of approximately 180° about the C4—C5 bond. The bond lengths and bond angles are found to have normal values (Allen *et al.*, 1987) and agree with a related structure (Patil *et al.*, 2006) with the execption of some parameters of the thiophene ring, which are probably a consequence of the disorder. The benzene and thiophene rings are individually planar, with maximum deviations of 0.019 (2) Å for atom C12 and -0.074 (2)Å for atom C1X. The molecule is twisted about the C7—C8 bond with a dihedral angle of 13.93 (19)° between the benzene ring and S1/C1—C4 [15.9 (19)° for S1X/C1X—C3X].

Intramolecular hydrogen bond C—H···O generates ring motif S(5)S(5) (Bernstein *et al.*, 1995). The crystal packing is stabilized by intra and intermolecular C—H···O interactions. π—π interactions between the S1/C1—C4 (centroid *Cg*1) and C8—C13 (centroid *Cg*3) rings [*Cg*1···*Cg*3^i^=3.852 (2) Å] [symmetry code: (i) -*x*,-*y*,-*z*] together with S···O=2.9378 (12)Å and O···O=2.5811 (16)Å short contacts are observed.

### Experimental

2,4,5-trimethoxybenzaldehyde (0.01 mol, 1.96 g m) in ethanol (30 ml) was mixed with 2-acetylthiophene (0.01 mol, 1.07 ml) in 30 ml ethanol and the mixture was treated with 10 ml of 10% sodium hydroxide solution and stirred at room temperature for 8 h. The precipitate obtained was poured into ice-cold water (500 ml) and left to stand for 5 h. The resulting crude solid was filtered, dried and recrystallized from N, *N*-dimethylformamide (DMF) by slow evaporation.

### Refinement

H atoms were positioned geometrically [C—H = 0.93Å; and CH_3_=0.96 Å] and refined using a riding model, with *U*_iso_(H) = 1.2*U*_eq_(C) and 1.5_eq_(C_methyl_). A rotating group model was used for the methyl groups. The ratio of the refined occupancies for the major and minor components of the disordered thiophene ring is 0.872 (2):0.128 (2). Similarity and rigid-bond restraints were applied to the disordered atoms.

### Crystal data

**Table:**

C_16_H_16_O_4_S	*F*_000_ = 640
*M**_r_* = 304.35	*D*_x_ = 1.401 Mg m^−^^3^
Monoclinic, *P*2_1_/*c*	Mo *K*α radiation λ = 0.71073 Å
Hall symbol: -P 2ybc	Cell parameters from 4291 reflections
*a* = 7.53910 (10) Å	θ = 2.7–28.4º
*b* = 7.92250 (10) Å	µ = 0.24 mm^−^^1^
*c* = 24.3399 (3) Å	*T* = 100.0 (1) K
β = 97.0210 (10)º	Block, yellow
*V* = 1442.88 (3) Å^3^	0.25 × 0.22 × 0.14 mm
*Z* = 4	

### Data collection

**Table:**

Bruker SMART APEXII CCD area-detector diffractometer	4256 independent reflections
Radiation source: fine-focus sealed tube	3174 reflections with *I* > 2σ(*I*)
Monochromator: graphite	*R*_int_ = 0.063
*T* = 100.0(1) K	θ_max_ = 30.1º
φ and ω scans	θ_min_ = 1.7º
Absorption correction: multi-scan(SADABS; Bruker, 2005)	*h* = −10→10
*T*_min_ = 0.942, *T*_max_ = 0.967	*k* = −11→10
33027 measured reflections	*l* = −34→34

### Refinement

**Table:**

Refinement on *F*^2^	Secondary atom site location: difference Fourier map
Least-squares matrix: full	Hydrogen site location: inferred from neighbouring sites
*R*[*F*^2^ > 2σ(*F*^2^)] = 0.046	H-atom parameters constrained
*wR*(*F*^2^) = 0.120	*w* = 1/[σ^2^(*F*_o_^2^) + (0.0532*P*)^2^ + 0.4695*P*] where *P* = (*F*_o_^2^ + 2*F*_c_^2^)/3
*S* = 1.07	(Δ/σ)_max_ < 0.001
4256 reflections	Δρ_max_ = 0.41 e Å^−^^3^
211 parameters	Δρ_min_ = −0.22 e Å^−^^3^
120 restraints	Extinction correction: none
Primary atom site location: structure-invariant direct methods	

### Special details

**Table:**

Experimental. The data was collected with the Oxford Cyrosystem Cobra low-temperature attachment.
Geometry. All e.s.d.'s (except the e.s.d. in the dihedral angle between two l.s. planes) are estimated using the full covariance matrix. The cell e.s.d.'s are taken into account individually in the estimation of e.s.d.'s in distances, angles and torsion angles; correlations between e.s.d.'s in cell parameters are only used when they are defined by crystal symmetry. An approximate (isotropic) treatment of cell e.s.d.'s is used for estimating e.s.d.'s involving l.s. planes.
Refinement. Refinement of *F*^2^ against ALL reflections. The weighted *R*-factor *wR* and goodness of fit *S* are based on *F*^2^, conventional *R*-factors *R* are based on *F*, with *F* set to zero for negative *F*^2^. The threshold expression of *F*^2^ > σ(*F*^2^) is used only for calculating *R*-factors(gt) *etc*. and is not relevant to the choice of reflections for refinement. *R*-factors based on *F*^2^ are statistically about twice as large as those based on *F*, and *R*- factors based on ALL data will be even larger.

### Fractional atomic coordinates and isotropic or equivalent isotropic displacement parameters (Å^2^)

**Table:**

	*x*	*y*	*z*	*U*_iso_*/*U*_eq_	Occ. (<1)
O1	0.21432 (18)	−0.07151 (14)	0.09237 (4)	0.0317 (3)	
O2	0.17726 (15)	0.05965 (14)	−0.21790 (4)	0.0241 (2)	
O3	0.32216 (15)	−0.21704 (14)	−0.24632 (4)	0.0245 (2)	
O4	0.38751 (16)	−0.41134 (14)	−0.05568 (4)	0.0256 (3)	
C4	0.1783 (2)	0.22121 (18)	0.08330 (6)	0.0207 (3)	
S1	0.17822 (8)	0.24407 (6)	0.153399 (18)	0.02221 (15)	0.870 (2)
C1	0.1475 (5)	0.4570 (4)	0.14728 (11)	0.0232 (6)	0.870 (2)
H1A	0.1413	0.5286	0.1773	0.028*	0.870 (2)
C2	0.1330 (9)	0.5112 (4)	0.09346 (15)	0.0270 (8)	0.870 (2)
H2A	0.1119	0.6223	0.0822	0.032*	0.870 (2)
C3	0.1544 (10)	0.3750 (7)	0.05759 (18)	0.0335 (12)	0.870 (2)
H3A	0.1526	0.3883	0.0196	0.040*	0.870 (2)
S1X	0.1377 (15)	0.3871 (9)	0.0437 (3)	0.0239 (13)	0.130 (2)
C1X	0.145 (7)	0.511 (3)	0.1014 (8)	0.036 (8)*	0.130 (2)
H1XA	0.1481	0.6281	0.1003	0.043*	0.130 (2)
C2X	0.147 (4)	0.421 (2)	0.1494 (9)	0.027 (6)*	0.130 (2)
H2XA	0.1296	0.4635	0.1839	0.032*	0.130 (2)
C3X	0.180 (3)	0.253 (2)	0.1369 (6)	0.0335 (12)	0.130 (2)
H3XA	0.2008	0.1699	0.1640	0.040*	0.130 (2)
C5	0.2048 (2)	0.05105 (19)	0.06134 (6)	0.0227 (3)	
C6	0.2186 (2)	0.03664 (19)	0.00174 (6)	0.0237 (3)	
H6A	0.1975	0.1315	−0.0206	0.028*	
C7	0.2608 (2)	−0.10914 (19)	−0.02109 (6)	0.0224 (3)	
H7A	0.2816	−0.2009	0.0027	0.027*	
C8	0.2776 (2)	−0.13959 (18)	−0.07931 (6)	0.0203 (3)	
C9	0.3410 (2)	−0.29444 (19)	−0.09632 (6)	0.0205 (3)	
C10	0.3580 (2)	−0.32485 (19)	−0.15198 (6)	0.0210 (3)	
H10A	0.4022	−0.4277	−0.1627	0.025*	
C11	0.3088 (2)	−0.20143 (19)	−0.19118 (6)	0.0200 (3)	
C12	0.2371 (2)	−0.04789 (19)	−0.17534 (6)	0.0204 (3)	
C13	0.2256 (2)	−0.01779 (19)	−0.12019 (6)	0.0211 (3)	
H13A	0.1823	0.0857	−0.1097	0.025*	
C14	0.0920 (2)	0.2100 (2)	−0.20310 (6)	0.0280 (4)	
H14A	0.0559	0.2753	−0.2358	0.042*	
H14B	0.1736	0.2746	−0.1780	0.042*	
H14C	−0.0113	0.1815	−0.1855	0.042*	
C15	0.3832 (2)	−0.3757 (2)	−0.26506 (7)	0.0305 (4)	
H15A	0.3869	−0.3710	−0.3043	0.046*	
H15B	0.3028	−0.4635	−0.2568	0.046*	
H15C	0.5007	−0.3988	−0.2467	0.046*	
C16	0.4083 (2)	−0.5814 (2)	−0.07198 (7)	0.0278 (3)	
H16B	0.4405	−0.6498	−0.0397	0.042*	
H16C	0.5006	−0.5879	−0.0958	0.042*	
H16D	0.2979	−0.6215	−0.0914	0.042*	

### Atomic displacement parameters (Å^2^)

**Table:**

	*U*^11^	*U*^22^	*U*^33^	*U*^12^	*U*^13^	*U*^23^
O1	0.0575 (8)	0.0197 (6)	0.0187 (5)	0.0009 (5)	0.0076 (5)	0.0019 (4)
O2	0.0354 (6)	0.0222 (5)	0.0150 (5)	0.0045 (5)	0.0046 (4)	0.0008 (4)
O3	0.0346 (6)	0.0249 (6)	0.0151 (5)	0.0023 (5)	0.0077 (4)	−0.0036 (4)
O4	0.0389 (6)	0.0205 (5)	0.0167 (5)	0.0049 (5)	0.0009 (4)	−0.0005 (4)
C4	0.0270 (7)	0.0213 (7)	0.0144 (6)	−0.0020 (6)	0.0050 (5)	−0.0017 (5)
S1	0.0312 (3)	0.0219 (2)	0.0139 (2)	−0.00063 (17)	0.0044 (2)	−0.00192 (18)
C1	0.0330 (13)	0.0168 (12)	0.0206 (11)	0.0017 (12)	0.0061 (7)	−0.0042 (9)
C2	0.0443 (19)	0.0168 (11)	0.0206 (11)	−0.0016 (8)	0.0072 (15)	−0.0015 (7)
C3	0.0466 (19)	0.0352 (17)	0.020 (2)	−0.0016 (12)	0.0106 (17)	−0.0004 (13)
S1X	0.041 (3)	0.0111 (18)	0.022 (3)	−0.0021 (16)	0.014 (2)	−0.0060 (17)
C3X	0.0466 (19)	0.0352 (17)	0.020 (2)	−0.0016 (12)	0.0106 (17)	−0.0004 (13)
C5	0.0312 (8)	0.0208 (7)	0.0167 (7)	−0.0011 (6)	0.0048 (6)	−0.0011 (5)
C6	0.0353 (8)	0.0201 (7)	0.0164 (7)	−0.0018 (6)	0.0058 (6)	0.0006 (5)
C7	0.0277 (8)	0.0234 (7)	0.0163 (6)	−0.0010 (6)	0.0025 (6)	−0.0002 (5)
C8	0.0269 (7)	0.0190 (7)	0.0153 (6)	−0.0026 (6)	0.0042 (5)	−0.0023 (5)
C9	0.0242 (7)	0.0203 (7)	0.0166 (7)	−0.0006 (6)	0.0005 (5)	−0.0002 (5)
C10	0.0256 (7)	0.0207 (7)	0.0172 (7)	0.0005 (6)	0.0042 (5)	−0.0033 (5)
C11	0.0228 (7)	0.0234 (7)	0.0140 (6)	−0.0023 (6)	0.0037 (5)	−0.0021 (5)
C12	0.0246 (7)	0.0207 (7)	0.0163 (6)	−0.0017 (6)	0.0039 (5)	0.0006 (5)
C13	0.0286 (8)	0.0184 (7)	0.0169 (7)	−0.0009 (6)	0.0047 (6)	−0.0022 (5)
C14	0.0395 (9)	0.0243 (8)	0.0201 (7)	0.0073 (7)	0.0030 (6)	0.0005 (6)
C15	0.0429 (10)	0.0298 (9)	0.0196 (7)	0.0075 (7)	0.0064 (7)	−0.0071 (6)
C16	0.0396 (9)	0.0207 (7)	0.0221 (7)	0.0019 (7)	−0.0002 (6)	−0.0007 (6)

### Geometric parameters (Å, °)

**Table:**

O1—C5	1.2268 (18)	C3X—H3XA	0.9300
O2—C12	1.3741 (17)	C5—C6	1.472 (2)
O2—C14	1.4206 (19)	C6—C7	1.337 (2)
O3—C11	1.3637 (17)	C6—H6A	0.9300
O3—C15	1.4319 (19)	C7—C8	1.4582 (19)
O4—C9	1.3692 (17)	C7—H7A	0.9300
O4—C16	1.4182 (18)	C8—C9	1.398 (2)
C4—C3X	1.328 (13)	C8—C13	1.407 (2)
C4—C3	1.371 (5)	C9—C10	1.3972 (19)
C4—C5	1.473 (2)	C10—C11	1.385 (2)
C4—S1X	1.636 (7)	C10—H10A	0.9300
C4—S1	1.7158 (15)	C11—C12	1.404 (2)
S1—C1	1.707 (3)	C12—C13	1.376 (2)
C1—C2	1.370 (4)	C13—H13A	0.9300
C1—H1A	0.9300	C14—H14A	0.9600
C2—C3	1.410 (6)	C14—H14B	0.9600
C2—H2A	0.9300	C14—H14C	0.9600
C3—H3A	0.9300	C15—H15A	0.9600
S1X—C1X	1.708 (18)	C15—H15B	0.9600
C1X—C2X	1.368 (17)	C15—H15C	0.9600
C1X—H1XA	0.9300	C16—H16B	0.9600
C2X—C3X	1.391 (17)	C16—H16C	0.9600
C2X—H2XA	0.9300	C16—H16D	0.9600
			
C12—O2—C14	116.47 (11)	C6—C7—H7A	116.6
C11—O3—C15	117.29 (12)	C8—C7—H7A	116.6
C9—O4—C16	117.89 (11)	C9—C8—C13	117.93 (13)
C3X—C4—C3	105.4 (8)	C9—C8—C7	120.43 (13)
C3X—C4—C5	123.0 (8)	C13—C8—C7	121.61 (13)
C3—C4—C5	131.6 (2)	O4—C9—C10	122.55 (13)
C3X—C4—S1X	113.9 (8)	O4—C9—C8	116.52 (12)
C5—C4—S1X	123.0 (3)	C10—C9—C8	120.92 (13)
C3—C4—S1	110.1 (2)	C11—C10—C9	119.84 (14)
C5—C4—S1	118.30 (11)	C11—C10—H10A	120.1
S1X—C4—S1	118.6 (3)	C9—C10—H10A	120.1
C1—S1—C4	91.96 (10)	O3—C11—C10	124.62 (13)
C2—C1—S1	112.8 (2)	O3—C11—C12	115.23 (13)
C2—C1—H1A	123.6	C10—C11—C12	120.15 (13)
S1—C1—H1A	123.6	O2—C12—C13	125.01 (13)
C1—C2—C3	110.5 (3)	O2—C12—C11	115.59 (12)
C1—C2—H2A	124.7	C13—C12—C11	119.36 (13)
C3—C2—H2A	124.7	C12—C13—C8	121.70 (14)
C4—C3—C2	114.6 (3)	C12—C13—H13A	119.2
C4—C3—H3A	122.7	C8—C13—H13A	119.2
C2—C3—H3A	122.7	O2—C14—H14A	109.5
C4—S1X—C1X	89.5 (9)	O2—C14—H14B	109.5
C2X—C1X—S1X	113.4 (16)	H14A—C14—H14B	109.5
C2X—C1X—H1XA	123.3	O2—C14—H14C	109.5
S1X—C1X—H1XA	123.3	H14A—C14—H14C	109.5
C1X—C2X—C3X	107.3 (17)	H14B—C14—H14C	109.5
C1X—C2X—H2XA	126.4	O3—C15—H15A	109.5
C3X—C2X—H2XA	126.4	O3—C15—H15B	109.5
C4—C3X—C2X	114.5 (14)	H15A—C15—H15B	109.5
C4—C3X—H3XA	122.8	O3—C15—H15C	109.5
C2X—C3X—H3XA	122.8	H15A—C15—H15C	109.5
O1—C5—C6	122.66 (14)	H15B—C15—H15C	109.5
O1—C5—C4	120.09 (13)	O4—C16—H16B	109.5
C6—C5—C4	117.25 (13)	O4—C16—H16C	109.5
C7—C6—C5	121.74 (14)	H16B—C16—H16C	109.5
C7—C6—H6A	119.1	O4—C16—H16D	109.5
C5—C6—H6A	119.1	H16B—C16—H16D	109.5
C6—C7—C8	126.89 (14)	H16C—C16—H16D	109.5
			
C3X—C4—S1—C1	13 (12)	S1—C4—C5—C6	−175.26 (11)
C3—C4—S1—C1	−0.2 (4)	O1—C5—C6—C7	−7.0 (3)
C5—C4—S1—C1	178.97 (18)	C4—C5—C6—C7	173.08 (15)
S1X—C4—S1—C1	−4.5 (5)	C5—C6—C7—C8	179.63 (14)
C4—S1—C1—C2	1.5 (4)	C6—C7—C8—C9	173.21 (16)
S1—C1—C2—C3	−2.2 (7)	C6—C7—C8—C13	−8.9 (3)
C3X—C4—C3—C2	−2.2 (11)	C16—O4—C9—C10	−16.9 (2)
C5—C4—C3—C2	179.9 (4)	C16—O4—C9—C8	164.24 (14)
S1X—C4—C3—C2	155 (5)	C13—C8—C9—O4	−178.88 (13)
S1—C4—C3—C2	−1.0 (7)	C7—C8—C9—O4	−0.9 (2)
C1—C2—C3—C4	2.1 (8)	C13—C8—C9—C10	2.2 (2)
C3X—C4—S1X—C1X	6(2)	C7—C8—C9—C10	−179.86 (14)
C3—C4—S1X—C1X	−18 (4)	O4—C9—C10—C11	−179.94 (14)
C5—C4—S1X—C1X	−175.8 (18)	C8—C9—C10—C11	−1.1 (2)
S1—C4—S1X—C1X	7.8 (19)	C15—O3—C11—C10	3.3 (2)
C4—S1X—C1X—C2X	−11 (4)	C15—O3—C11—C12	−175.89 (14)
S1X—C1X—C2X—C3X	12 (5)	C9—C10—C11—O3	179.05 (13)
C3—C4—C3X—C2X	4(2)	C9—C10—C11—C12	−1.8 (2)
C5—C4—C3X—C2X	−178.2 (17)	C14—O2—C12—C13	−2.3 (2)
S1X—C4—C3X—C2X	0(3)	C14—O2—C12—C11	175.38 (14)
S1—C4—C3X—C2X	−163 (13)	O3—C11—C12—O2	4.99 (19)
C1X—C2X—C3X—C4	−8(4)	C10—C11—C12—O2	−174.20 (13)
C3X—C4—C5—O1	6.3 (12)	O3—C11—C12—C13	−177.23 (13)
C3—C4—C5—O1	−176.2 (4)	C10—C11—C12—C13	3.6 (2)
S1X—C4—C5—O1	−171.6 (5)	O2—C12—C13—C8	175.11 (14)
S1—C4—C5—O1	4.8 (2)	C11—C12—C13—C8	−2.4 (2)
C3X—C4—C5—C6	−173.8 (12)	C9—C8—C13—C12	−0.4 (2)
C3—C4—C5—C6	3.7 (5)	C7—C8—C13—C12	−178.33 (14)
S1X—C4—C5—C6	8.3 (5)		

### Hydrogen-bond geometry (Å, °)

**Table:**

*D*—H···*A*	*D*—H	H···*A*	*D*···*A*	*D*—H···*A*
C2—H2A···O1^i^	0.93	2.55	3.363 (4)	146
C7—H7A···O1	0.93	2.52	2.8416 (18)	101
C7—H7A···O4	0.93	2.39	2.7477 (19)	103
C16—H16B···O4^ii^	0.96	2.56	3.2952 (19)	133

Symmetry codes: (i) *x*, *y*+1, *z*; (ii) −*x*+1, −*y*−1, −*z*.

## References

[bb1] Allen, F. H., Kennard, O., Watson, D. G., Brammer, L., Orpen, A. G. & Taylor, R. (1987). *J. Chem. Soc. Perkin Trans. 2*, pp. S1–19.

[bb2] Bernstein, J., Davis, R. E., Shimoni, L. & Chang, N. L. (1995). *Angew. Chem. Int. Ed. Engl.* **34**, 1555–1573.

[bb3] Bruker (2005). *APEX2*, *SAINT* and *SADABS* Bruker AXS Inc., Madison, Wisconsin, USA.

[bb4] Chantrapromma, S., Jindawong, B., Fun, H.-K., Anjum, S. & Karalai, C. (2005). *Acta Cryst.* E**61**, o2096–o2098.

[bb5] Chantrapromma, S., Ruanwas, P., Jindawong, B., Razak, I. A. & Fun, H.-K. (2006). *Acta Cryst.* E**62**, o875–o877.

[bb6] Fun, H.-K., Rodwatcharapiban, P., Jindawong, B. & Chantrapromma, S. (2006). *Acta Cryst.* E**62**, o2725–o2727.

[bb7] Patil, P. S., Dharmaprakash, S. M., Ramakrishna, K., Fun, H.-K., Sai Santosh Kumar, R. & Rao, D. N. (2007). *J. Cryst. Growth*, **303**, 520–524.

[bb8] Patil, P. S., Fun, H.-K., Chantrapromma, S. & Dharmaprakash, S. M. (2007). *Acta Cryst.* E**63**, o2497–o2498.

[bb9] Patil, P. S., Ng, S.-L., Razak, I. A., Fun, H.-K. & Dharmaprakash, S. M. (2006). E**62**, o3718–o3720.

[bb10] Sheldrick, G. M. (2008). *Acta Cryst.* A**64**, 112–122. 10.1107/S0108767307043930 18156677

[bb11] Spek, A. L. (2003). *J. Appl. Cryst.* **36**, 7–13.

